# Supplementary material for: Hybrid Models and Biological Model Reduction with PyDSTool
Source: PLoS Comput Biol. 2012 Aug 9;8(8):e1002628. doi: 10.1371/journal.pcbi.1002628 (PMC3415397; doi:10.1371/journal.pcbi.1002628)
Supplement: Text S4 — Complete source code for the PyDSTool package (version 0.88.120504). Includes API documentation and help files linking to web pages. This file is identical to the current public release on Sourceforge.net. (ZIP) [file pcbi.1002628.s004.zip › PyDSTool/html/PyDSTool.Toolbox.mechmatlib-pysrc.html]

xml version="1.0" encoding="ascii"?


PyDSTool.Toolbox.mechmatlib


| Home | Trees | Indices | Help | PyDSTool |
| --- | --- | --- | --- | --- |

Package PyDSTool ::
Package Toolbox ::
Module mechmatlib

[hide private]

[frames] | no frames]

# Source Code for Module PyDSTool.Toolbox.mechmatlib

```
  1  # Library functions for building matrices for rigid body mechanics
 
  2  # Robert Clewley, (c) 2005, 2006
 
  3  
 
  4  from numpy import mat, identity, shape, resize, dot, array, transpose 
  5  import math 
  6  import copy 
  7  
 
  8  __all__ = ['augment_3x3_matrix', 'augment_3_vector', 'ZeroPos', 'Id',
 
  9             'make_rot', 'make_disp', 'make_T', 'cross', 'dot'] 
 10  
 
 11  # ---------------------------------------------------------------------------
 
 12  
 


13 -def augment_3_vector(v=array([0., 0., 0.]), free=False):


14      if free: 
 15          freeval = 0. 
 16      else: 
 17          freeval = 1. 
 18      assert shape(v) == (3,), "v argument must be 3-vector -- found "+str(shape(v)) 
 19      vaug = resize(v,(4,)) 
 20      vaug[3] = freeval 
 21      return vaug

 22  
 
 23  ZeroPos = augment_3_vector([0., 0., 0.]) 
 24  
 


25 -def augment_3x3_matrix(M=mat(identity(3,'f')),
 
 26                     disp=array([0., 0., 0.]), free=False):


27      if free: 
 28          freeval = 0. 
 29      else: 
 30          freeval = 1. 
 31      assert shape(M) == (3,3), "M argument must be 3 x 3 matrix" 
 32      assert shape(disp) == (3,), "displacement argument must be 1 x 3 array" 
 33      # resize works properly only on rows ... therefore,
 
 34      # use transpose twice. (resize on columns messes up matrix contents)
 
 35      Mxr = mat(resize(M.array, (4,3))) 
 36      Mxr[3,:] = [0.,0.,0.] 
 37      MxrcT = mat(resize((Mxr.T).array, (4,4))) 
 38      try: 
 39          # array
 
 40          dcol = copy.copy(disp).resize(4) 
 41          dcol[3] = freeval 
 42      except AttributeError: 
 43          # list
 
 44          dcol = copy.copy(disp) 
 45          dcol.append(freeval) 
 46      MxrcT[3,:] = dcol 
 47      return MxrcT.T

 48  
 
 49  
 


50 -def make_disp(r):


51      """Make 3D displacement
 
 52      """ 
 53      return augment_3x3_matrix(disp=r)

 54  
 
 55  
 


56 -def make_rot(a):


57      """Make 3D rotation (around z, then new x, then new y)
 
 58      """ 
 59      a1 = a[0] 
 60      a2 = a[1] 
 61      a3 = a[2] 
 62      
 
 63      ca1 = math.cos(a1) 
 64      sa1 = math.sin(a1) 
 65      ca2 = math.cos(a2) 
 66      sa2 = math.sin(a2) 
 67      ca3 = math.cos(a3) 
 68      sa3 = math.sin(a3) 
 69      
 
 70      # rot: new y, by a3
 
 71      roty = augment_3x3_matrix(mat([[ca3, 0., sa3], [0., 1., 0.],
 
 72                                                [-sa3, 0, ca3]])) 
 73  
 
 74      # rot: new x, by a2
 
 75      rotx = augment_3x3_matrix(mat([[1., 0., 0.], [0., ca2, -sa2],
 
 76                                                [0., sa2, ca2]])) 
 77  
 
 78      # rot: z, by a1
 
 79      rotz = augment_3x3_matrix(mat([[ca1, -sa1, 0.], [sa1, ca1, 0.],
 
 80                                                [0., 0., 1.]])) 
 81      return roty*rotx*rotz

 82  
 
 83  
 


84 -def make_T(r, a):


85      """Make general 3D transformation (displacement then rotation)
 
 86      """ 
 87      R = make_rot(a) 
 88      D = make_disp(r) 
 89      return R*D

 90  
 
 91  # Identity matrix
 
 92  Id = mat(identity(4,'f')) 
 93  
 


94 -def cross(a,b):


95      """Cross product of two 3D vectors
 
 96      """ 
 97      c = array([0., 0., 0.]) 
 98      c[0] = a[1]*b[2]-a[2]*b[1] 
 99      c[1] = a[2]*b[0]-a[0]*b[2] 
100      c[2] = a[0]*b[1]-a[1]*b[0] 
101      return c

102
```

  


| Home | Trees | Indices | Help | PyDSTool |
| --- | --- | --- | --- | --- |

|  |  |
| --- | --- |
| Generated by Epydoc 3.0.1 on Fri May 4 15:24:25 2012 | http://epydoc.sourceforge.net |
